# Diagnostic Significance of Renal Artery Resistive Index (RRI), Caudal Vena Cava Diameter (CVC), and Aorta (Ao) in Dogs with Hypovolemia

**DOI:** 10.3390/vetsci13040402

**Published:** 2026-04-19

**Authors:** Ghada Ashraf, Mohamed Marzok, Sabry El-khodery, Al Mohamad Zakriya, Adel Almubarak, Mohammed Albaloushi, Mohamed Ali

**Affiliations:** 1 Department of Internal Medicine and Infectious Diseases, Faculty of Veterinary Medicine, Badr University in Cairo (BUC), Badr City 32897, Egypt; ghada.ashraf@buc.edu.eg; 2 Department of Clinical Sciences, College of Veterinary Medicine, King Faisal University, Al-Ahsa 31982, Saudi Arabia; 3 Department of Internal Medicine and Infectious Diseases, Faculty of Veterinary Medicine, Mansoura University, Mansoura 35516, Egypt; 4 Hospital Job Dental Clinic Complex, King Faisal University, Al-Ahsa 31982, Saudi Arabia; 5 Department of Internal Medicine and Infectious Diseases, Faculty of Veterinary Medicine, Cairo University, Giza 12211, Egypt

**Keywords:** BUN, cardiovascular, canine, CBC, dehydration, diagnostic value

## Abstract

Hypovolemia is a systemic state characterized by severe reduction in the effective blood volume with subsequent tissue hypoperfusion. It may be due to fluid loss, decreased water intake, fluid redistribution, or systemic disease. The present study aimed to evaluate the diagnostic significance of the renal artery resistive index (RRI), caudal vena cava (CVC) diameter, and aorta (Ao) diameter in dogs with hypovolemia. For this purpose, 30 dogs (hypovolemic, *n* = 15; control, *n* = 15) were investigated. Clinical variables and hematological findings were investigated for each dog. Pulsed-wave Doppler ultrasound was performed to measure the RRI and diameters of the CVC and Ao. Ultrasound examination revealed a markedly elevated RRI (*p* < 0.001) and significantly reduced CVC (*p* < 0.001) and Ao (*p* < 0.001) diameters in hypovolemic dogs compared to controls, reflecting increased vascular resistance and impaired venous return. Biochemical analysis showed significant increases in blood urea nitrogen (BUN) and BUN:Cr ratio, while Cr remained unchanged. Hematological variables demonstrated limited diagnostic value, with only mild increases in packed cell volume (PCV%). Correlation analysis confirmed a strong positive correlation between RRI and BUN (r = 0.917; *p* < 0.01), RRI and BUN:Cr ratio (r = 0.664; *p* < 0.01), and CVC and Ao diameters (r = 0.832; *p* < 0.05). Receiver operating characteristic (ROC) analysis with area under the curve (AUC) identified RRI (AUC = 0.99), CVC diameter (AUC = 0.93), and Ao diameter (AUC = 0.88) as highly accurate markers of hypovolemia, whereas the CVC:Ao ratio and hematological markers provided poor discrimination. Logistic regression confirmed significant diagnostic value for RRI, CVC diameter, Ao diameter, and BUN, but final multivariate analysis revealed RRI as the sole independent early diagnostic marker (*p* < 0.001; OR: 196.0; 95% CI: 11.12–34.72). In conclusion, RRI measured by Doppler ultrasound is the most reliable and sensitive early diagnostic marker for hypovolemia in dogs, outperforming conventional biochemical and hematological markers.

## Simple Summary

The purpose of the present investigation was to assess the diagnostic value of the renal artery resistive index (RRI), diameter of the caudal vena cava (CVC), and aorta (Ao) diameter via pulsed-wave Doppler ultrasound in dogs with hypovolemia. For this purpose, 30 mongrel dogs (15 control and 15 with hypovolemia) were studied. Ultrasound examination revealed a marked increase in RRI (*p* < 0.001) and significant reduction in CVC (*p* < 0.001) and Ao (*p* < 0.001) diameters in hypovolemic dogs compared to controls, reflecting increased vascular resistance and impaired venous return. Biochemical analysis showed significant increases in blood urea nitrogen (BUN) and BUN:Cr ratio, while creatinine (Cr) remained unchanged. Logistic regression confirmed significant diagnostic value for the RRI, CVC diameter, Ao diameter, and BUN, but final multivariate analysis revealed RI as the sole independent early diagnostic marker (*p* < 0.001; OR: 196.0; 95% CI: 11.12–34.72). In conclusion, RRI measured by Doppler ultrasound is the most reliable and sensitive early diagnostic marker for hypovolemia in dogs, outperforming conventional biochemical and hematological markers.

## 1. Introduction

Hypovolemia is a common life-threatening clinical condition in small animals presented to emergency veterinarians [1]. It usually occurs as a result of a deleterious reduction in the effective blood volume, with subsequent development of hypovolemic shock [2,3]. Hypovolemia can exert critical effects on multiple body systems, such as the cardiovascular, respiratory, renal, and nervous systems [4]. It reduces blood volume, which in turn decreases blood pressure, pulse rate, and capillary refill time [5]. Early diagnosis and treatment of abnormal tissue perfusion are crucial for optimizing outcomes [6]. Clinicians should be familiar with the disease being treated and the types of fluids that are available [1].

A diagnosis of hypovolemia is usually based primarily on a clinical assessment of patient dogs [7,8]. Clinical evaluation involves the assessment of mucous membrane color, capillary refill time (CRT), pulse quality, mentation, heart rate, and body temperature [9,10]. However, the use of clinical variables to evaluate hypovolemia is usually subjective and not entirely accurate.

As a reduction in blood perfusion leads to several hemodynamic and body system responses [11], the detection of hematological and biochemical changes is recommended for diagnosis [12]. In hypovolemic dogs, PCV% and hemoglobin level are proposed as diagnostic markers, especially when hypovolemia is due to hemorrhage [13,14]. In addition, in clinically dehydrated dogs, total protein and albumin are significantly higher than in euhydrated dogs and rise with increasing dehydration grade, making them consistent biochemical indicators of dehydration [12]. Moreover, in critically ill dogs, precise measurement of intravascular volume and fluid responsiveness is essential. Despite their frequent interchangeability, these parameters are different but connected [15]. Therefore, measurement of traditional central venous pressure (CVP) via a central venous catheter has been used to evaluate hypovolemia [16]. However, all invasive techniques are time-consuming and may have associated risks. Noninvasive diagnostic techniques have been established in human medicine for the recognition of hypovolemia and assessment of response to resuscitation [17]. In veterinary medicine, the evaluation of hemodynamics using Doppler ultrasound is the most recent reliable technique for the evaluation of hypovolemia [18,19]. Ultrasonographic markers include the renal artery resistive index (RRI) [20], caudal vena cave diameter (CVC) [21], Ao diameter, and CVC:Ao ratio [22].

In dogs, RRI is best validated for identifying intrinsic renal disease; its use for hypovolemia remains speculative [23,24,25]. However, in cats, experimental data support the use of RRI trends to monitor changes in renal perfusion with hypotension or resuscitation [26,27]. In experimentally induced hypovolemia in dogs, there was a gradual increase in RRI proportional to blood loss [28]. The CVC diameter was found to decrease with collapse and a decrease in transmural pressure with intravascular volume loss. However, the extent of this decrease should be related to the Ao diameter and application of the CVC:Ao ratio for volume assessment [15].

The use of pulsed-Doppler ultrasound to assess RRI and the diameter of the CVC and Ao for the diagnosis of experimentally induced hypovolemia is frequent; however, its use in dogs with hypovolemia under field conditions is limited. Consequently, the present study aimed to assess the most acceptable diagnostic markers among ultrasound and hematobiochemical variables in dogs with hypovolemia.

## 2. Materials and Methods

### 2.1. Ethical Approval

This study was approved by the Ethical Committee of the Faculty of Veterinary Medicine, Cairo University (Vet CU13102024999). Before examining each dog, written consent was obtained from the owner.

### 2.2. Dogs

Thirty mongrel dogs were enrolled in this investigation with written consent from their owner and ethical approval. Dogs who were presented at the veterinary teaching hospital, Faculty of Veterinary Medicine, Cairo University, and a private shelter in Cairo city during the period from September 2022 to July 2024 with hypovolemia were included in this prospective observational study. Dogs had 3–20 kg of body weight (12.8 ± 3.4) and were 1–7 years of age (2.7 ± 1.2). There were eighteen male dogs included 12 neutered (hypovolemic, *n* = 5; control, *n* = 7) and 6 intact (hypovolemic, *n* = 2; control, *n* = 4). The remaining 12 females included 9 spayed (hypovolemic, *n* = 8; control, *n* = 1) and 3 intact (control). All animals under investigation were fed on either dry food (4 hypovolemic and 3 control), or homemade food (11 hypovolemic and 12 control). All dogs received the standard vaccination and deworming practice. Dogs were allocated into two groups: group 1, control group (*n* = 15); and group 2, hypovolemic group (*n* = 15). The main historical signs for ill dogs were diarrhea and vomiting for 1–3 days. Healthy control dogs were selected based on the history of an integrated vaccination program, regular receipt of anthelmintic drugs, and results of clinical and hematobiochemical investigations.

### 2.3. Selection Criteria

Dogs that fulfilled the inclusion criteria were those suffering from dehydration or hypovolemia, without signs of organ failure. Consequently, dogs with renal, heart, or hepatic failure with hypovolemia were excluded.

### 2.4. Clinical Examination

In accordance with the guidelines of the American Animal Hospital Association (AAHA), a clinical examination was performed for each dog [29]. A competent physical examination included measurement of heart rate, pulse rate, peripheral blood pressure (BP), respiratory rate, and rectal temperature. Evaluation of capillary refill time (CRT), mucosal membrane, and skin turgor were also performed for each animal. Dogs with a BP < 90 mmHg, CRT > 3 s, skin fold test > 10 s and Hypothermia (rectal temperature < 37.2 °C) were included in the study.

### 2.5. Whole-Blood and Serum Samples

For CBC analysis (RBCS count, hemoglobin content, PCV%, and total and differential leukocytic count), whole-blood samples were obtained from each dog and drawn from cephalic veins into EDTA-coated tubes (Euromed Co., Ltd., Cairo, Egypt). Additional whole-blood samples were obtained from each animal into plain test tubes. The blood was allowed to clot and centrifuged at 3000 rpm for five minutes. Clear non-hemolyzed sera were obtained and kept at −80 °C until analysis. Serum samples were used for measuring blood urea nitrogen (BUN), Cr, and serum total protein (TP). Blood sampling was performed, and further ultrasound examinations were conducted without administering any anesthesia.

### 2.6. Vascular Ultrasound

For ultrasound examination, all dogs were clipped over the area of the abdomen, and the skin was cleaned with alcohol before adding ultrasound gel. Ultrasound examination was performed using a 5–8 MHz micro convex probe (Edan AX8^®^ portable ultrasound). According to standard techniques [22,28], the dog was placed on the right lateral side. The probe was applied to the left mid-abdomen, just caudal to the last rib. Using the B-mode ultrasound, the transducer rotated slightly dorsally and cranially to obtain an ideal view. After identification of the renal hilum, the color Doppler mode was displayed to visualize blood flow. In Doppler evaluation of renal blood flow, the sagittal plane through the renal hilum is generally considered optimal. This plane allows for clear identification of the renal artery as it enters the hilum and facilitates alignment of the Doppler beam with the direction of blood flow, enabling more accurate color Doppler mapping and pulsed-wave Doppler spectral analysis. Doppler gain was adjusted during each examination to optimize visualization of blood flow while avoiding color noise artifacts. The pulsed-wave Doppler sample volume (gate) was adjusted according to the diameter of the evaluated vessel. In our protocol, the sample gate was kept small and positioned within the center of the vascular lumen to avoid contamination from adjacent vessels or surrounding tissue. Pulsed-wave Doppler was used to visualize and measure the RRI. The probe was then slid caudally until a view of the CVC and Ao at the aortic bifurcation was obtained. The following variables were measured: RRI, diameter of the CVC, diameter of the Ao, and CVC:Ao ratio [30]. The CVC and Ao diameters were measured during the expiratory phase to obtain stable hemodynamic conditions and exclude the effects of respiratory collapse. For reproducibility, two professional researchers were responsible for obtaining echocardiographic measurements. Each researcher recorded their own measurements and the interobserver coefficient of variation was recorded for each variable. Calculation of RRI was performed according to the following equation:

RRI = PSV − EDV/PSV.

where PSV is the peak systolic velocity and EDV is the end-diastolic velocity.

### 2.7. Statistical Analysis

Data were subjected to the Shapiro–Wilk test to determine evidence of normality distribution. The results were not significant, confirming the normal distribution of the data. Therefore, the data are presented as the mean and standard deviation. An unpaired *t*-test was used to detect variations between normal and hypovolemic dogs. An unpaired *t*-test was used also to detect the interobserver variability for the echocardiographic measurements. Pearson correlation was applied to assess the correlation among variables. Both ultrasonographic and hematological data for control and hypovolemic dogs were analyzed using receiver operating characteristic curve analysis to assess the diagnostic value of such variables. The results were presented as area under the curve, cut-off point, sensitivity, specificity, *p*-value, confidence interval at 95% (CI at 95%), and likelihood ratio. Logistic regression was performed to define the variables with the most reliable diagnostic value. In the first step, binary logistic regression was conducted, in which the dependent variable was the state of the dog (hypovolemic or not) and the independent variables were the diagnostic markers (ultrasound and hematological variables). Significant variables were further subjected to stepwise multivariate logistic regression models. The results of logistic regression analysis were presented as regression coefficients, *p*-values, confidence intervals (CIs at 95%), and odds ratios (ORs). For all analyses, a result was considered significant at *p* < 0.05. Data analysis was performed using a commercial software program (GraphPad Prism ver. 9.2, San Diego, CA, USA). However, logistic regression analysis was performed using the IBM_SPSS software ver. 21, New York, NY, USA.

## 3. Results

Thirty dogs were included in this investigation. Of these, 15 were hypovolemic and 15 were normal controls. Clinically, hypovolemic dogs showed a significant increase in heart rate (160 ± 21 vs. 120 ± 17; *p* < 0.01), respiratory rate (37 ± 6 vs. 25 ± 7; *p* < 0.01), and CRT (4 vs. 1; *p* < 0.01). However, there was a significant decrease in BP (88 ± 11 vs. 115 ± 9; *p* < 0.01). Pale mucous membranes were evident in most cases (14/15). Moreover, a weak pulse was evident in all cases.

**Figure 1:**
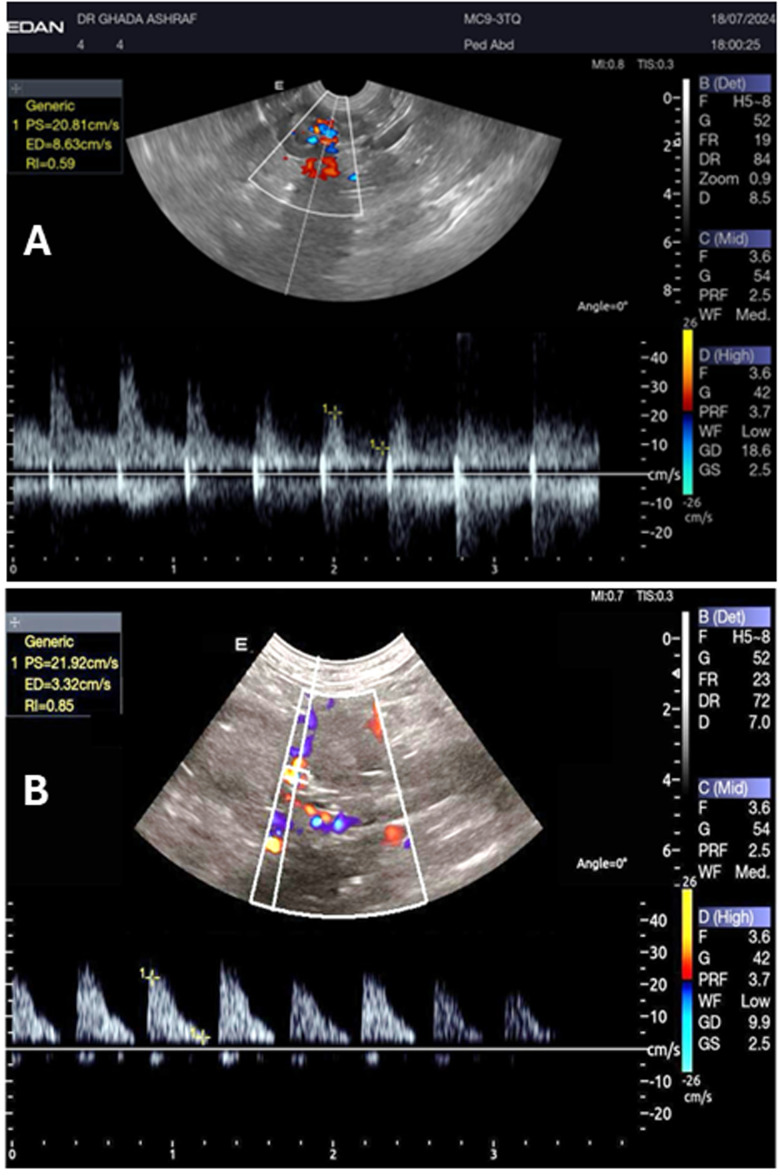
A pulsed wave Doppler image of the left renal artery in a two−year−old dog with normovolemia and RRI of 0.46 (**A**), and a dog with hypovolemia and RRI of 0.85 (**B**).

**Figure 2:**
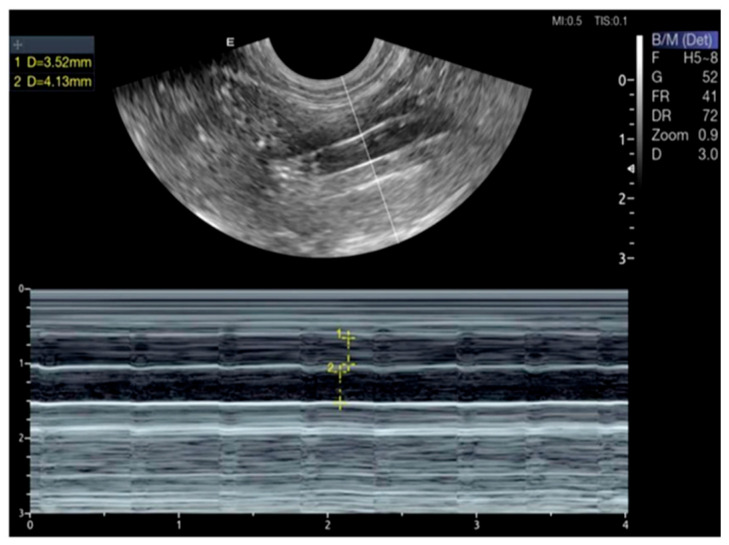
M-mode ultrasound of CVC and Ao of a 2-year-old dog with no signs of dehydration. M-mode was applied on CVC and Ao in the iliac position at aortic bifurcation, with a CVC:Ao ratio of 1.3.

**Figure 3:**
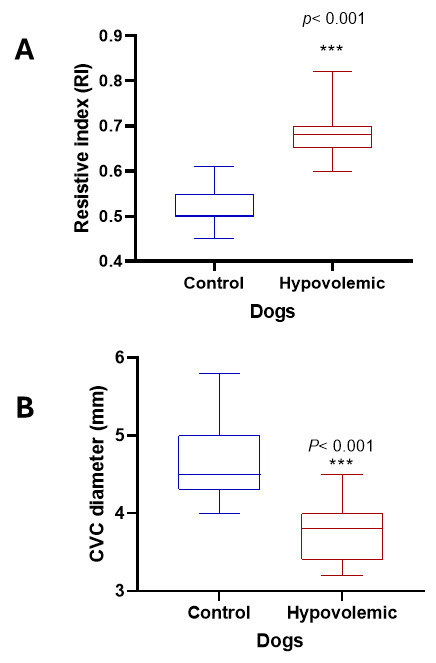
(**A**) Renal artery resistive index; (**B**) CVC diameter in normal dogs and in dogs with hypovolemia.***: *p* < 0.001.

**Figure 4:**
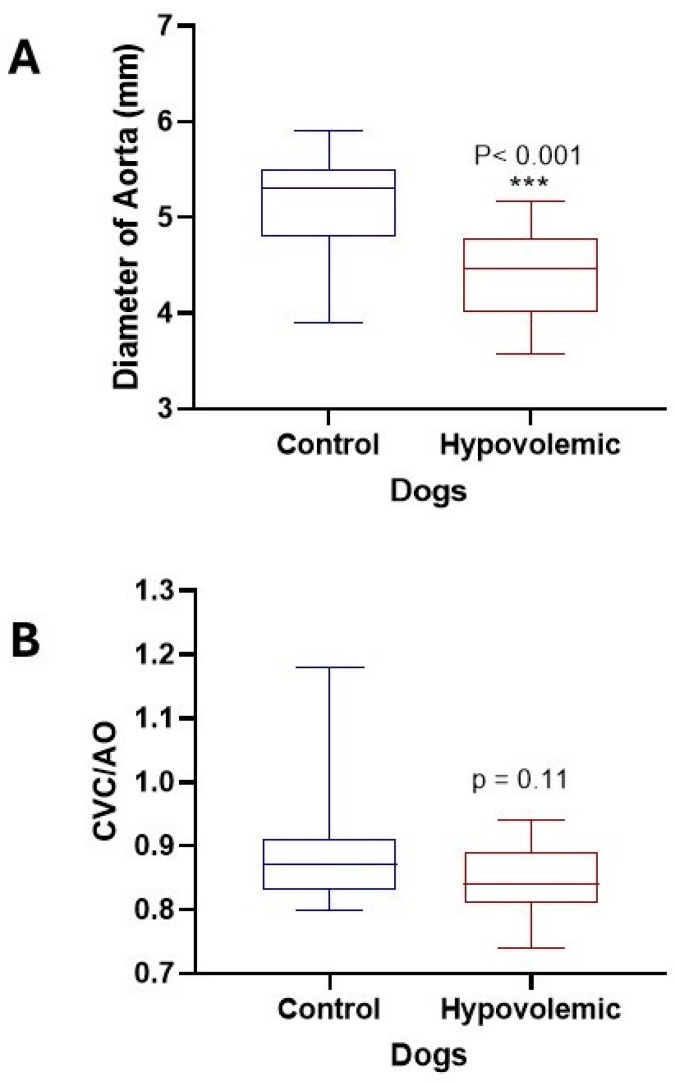
Diameter of Ao and CVC:Ao ratio in normal dogs and in dogs with hypovolemia. (**A**) Aortic diameter (mm); (**B**) CVC:Ao ratio. ***: *p* < 0.001.

Pulsed-wave Doppler ultrasound can visualize and measure the RRI and the diameter of the CVC and Ao (Figure 1 and Figure 2). On ultrasound examination, the RRI was markedly elevated (*p* < 0.001), and the diameter of the CVC was significantly reduced (*p* < 0.001), reflecting increased vascular resistance and reduced venous return (Figure 3). Moreover, the Ao diameter and the CVC:Ao ratio were significantly decreased in hypovolemic dogs compared with normal controls (Figure 4). Reproducibility of echocardiographic measurements was evidenced by the non-significant variation in the interobserver CV% for RRI (12.14 vs. 10.41), CVC diameter (10.65 vs. 11.25), and Ao diameter (10.13 vs. 9.74).

**Figure 5:**
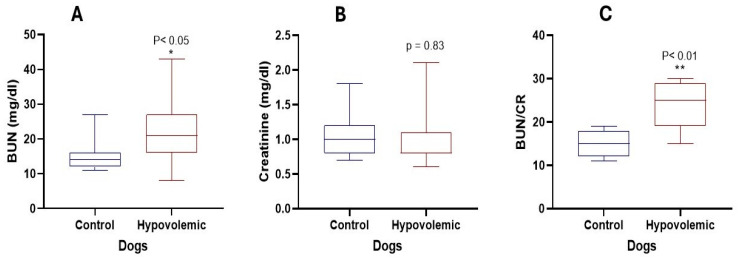
BUN, Cr and BUN:Cr ratio in normal dogs and in dogs with hypovolemia.(**A**) Blood urea nitrogen (BUN, mg/dL); (**B**) creatinine (Cr, mg/dL); (**C**) BUN:Cr ratio. *: *p* < 0.05; **: *p* < 0.01.

**Figure 6:**
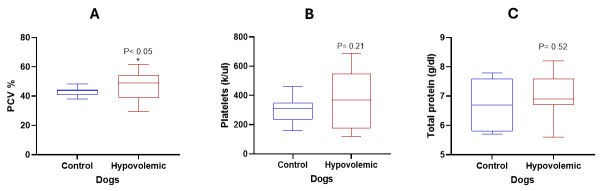
PCV%, platelets, and total protein in normal dogs and in dogs with hypovolemia. (**A**) PCV (%); (**B**) platelets (K/μL); (**C**) total protein (g/dL). *: *p* < 0.05.

Figure 5 shows the variations in renal function tests between normal and hypovolemic dogs. Dogs with hypovolemia showed a significant increase in BUN (*p* < 0.05) and BUN:Cr ratio (*p* < 0.01). However, Cr levels remained unchanged (*p* = 0.08). PCV % was mildly higher in hypovolemic dogs than in controls, but other hematological variables did not change significantly (Figure 6).

The RRI showed a strong positive correlation with BUN (r = 0.917; *p* < 0.01), BUN/CR ratio (r = 0.664; *p* < 0.01), and BUN and BUN/CR ratio (r = 0.761; *p* < 0.01). There was also a strong positive correlation between CVC and Ao diameter (r = 0.832; *p* < 0.05). Moreover, a moderate positive correlation was observed between TP and platelets (r = 0.589; *p* < 0.05). However, there was a negative correlation between CR and BUN/CR ratio (r = −0.642; *p* < 0.05). The remaining variables showed poor correlations.

**Figure 7:**
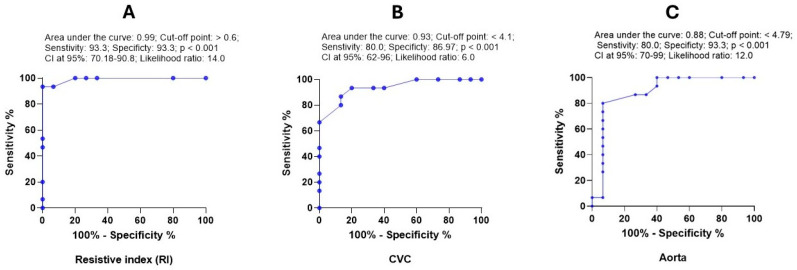
ROC curve for the diagnostic value of RRI (**A**), CVC diameter (**B**) and diameter of Ao (**C**) in dogs with hypovolemia. The curve shows the area under the curve (AUC), cut-off point, sensitivity, specificity, *p*-value, CI at 95% and likelihood ratio.

ROC analysis showed that RRI, CVC diameter, and Ao diameter were strong diagnostic markers for hypovolemia. The AUC values for these variables were 0.99, 0.93, and 0.88, respectively. Thus, these variables had high sensitivity and specificity for the detection of hypovolemia (Figure 7). However, CVC:Ao ratio provided poor non-significant prediction values (AUC: 0.62; *p* = 0.25).

**Figure 8:**
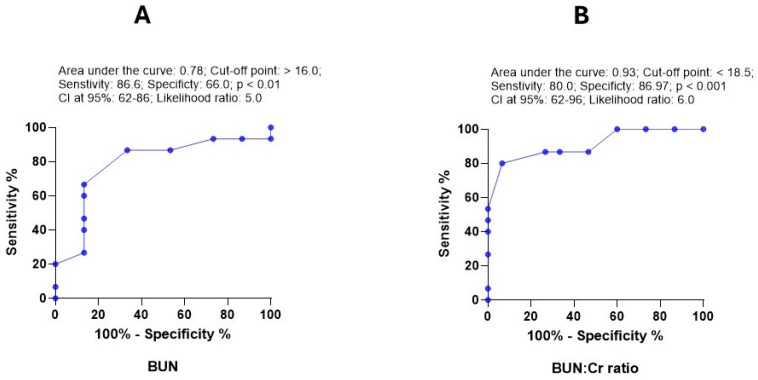
ROC curve for the diagnostic value of BUN (**A**) and BUN:Cr ratio (**B**) in dogs with hypovolemia. The curve shows the area under the curve (AUC), cut-off point, sensitivity, specificity, *p*-value, CI at 95% and likelihood ratio.

Regarding renal markers, Cr and BUN:Cr ratio had the highest AUC (0.93), followed by BUN (0.88). The sensitivity and specificity values varied (Figure 8). However, Cr provided poor diagnostic value. Hematological variables collectively showed poor accuracy in discriminating between normal and hypovolemic dogs. PCV% showed only mild diagnostic significance.

Univariate logistic regression analysis revealed that RRI, CVC diameter, Ao diameter, and serum BUN level had significant diagnostic values for the recognition of hypovolemia in dogs (Table 1). The variables with significant diagnostic values were RRI (*p* < 0.01; OR: 196.0; CI at 95%: 11.12–34.72), CVC diameter (*p* < 0.01; OR: 51.0; CI at 95%: 5.2–61.8), diameter of the Ao (*p* < 0.01; OR: 55.1; CI at 95%: 5.1–60.1), and serum level of BUN (*p* < 0.01; OR: 13.0; CI at 95%: 2.074–81.5). However, the final multivariate logistic regression model showed that RRI was the only significant diagnostic marker for hypovolemia in dogs (*p* < 0.001; OR: 196.0; CI at 95%: 11.12–34.7).

As shown in Table 2, the multivariate logistic regression model reveals that RRI was the only variable that provided significant early diagnostic prediction in dogs with hypovolemia (*p* < 0.001; OR: 196.0; CI at 95%: 11.12–34.72).

## 4. Discussion

Hypovolemia reduces circulating blood volume, which adversely affects renal and systemic hemodynamics [31]. The resistive indices of the renal artery (RIs), along with the diameters of the CVC and Ao, are ultrasonographic markers currently being used to assess volume status and fluid responsiveness in veterinary practice [6,31]. To the best of our knowledge, previous studies have evaluated renal and systemic hemodynamic changes in dogs with hypovolemia under experimental conditions. Therefore, the objective of the present study was to assess potential diagnostic ultrasound and hematological markers for hypovolemia in dogs under natural disease conditions.

Clinically, the present results showed that all examined dogs showed signs of dehydration and hypovolemia. The significant increase in HR, RR, and CRT with a decrease in BP may be caused by hypotension associated with a severe reduction in circulating blood volume. Similar findings were reported in experimental studies [22,28]. In hemorrhagic hypovolemia, it has been proposed that renal blood flow increases transiently due to a reduction in circulating blood volume, with subsequent vasoconstriction of renal blood vessels and a decrease in BP [28]. These findings were confirmed by an increase in PCV%, BUN levels, and BUN:Cr ratio. The results of the hematological analysis were in accordance with those of previous studies on diarrhea and hypovolemia in dogs [32,33]. However, Cr levels may remain unchanged between control dogs and those with renal impairment because Cr only increases after a significant loss of renal function, and factors, such as muscle mass, hydration, and compensatory renal mechanisms, can mask changes [34]. Some studies depend on the estimation of blood lactate as an indicator of hypovolemia [22]. Unfortunately, in the present study, we did not rely on the estimation of blood lactate because some studies have indicated that there are two types of hyperlactemia (A and B), in which type A is mainly affected by hypovolemia [35]. It has been stated that mild–moderate dehydration may show increased urine specific gravity and possibly kidney injury biomarkers, while BUN and Cr remain within reference limits, illustrating their limited sensitivity for early renal effects of hypovolemia [36].

Descriptive statistics revealed a significant increase in the RRI and a significant decrease in CVC and Ao diameters in hypovolemic dogs compared to the control group. Univariate logistic regression analysis revealed that the RRI, CVC diameter, Ao diameter, and BUN were significant diagnostic markers. However, multivariate analysis showed that only the RRI was the sole marker. This finding was supported by the results of the ROC analysis, which indicated excellent AUC (0.99), very high sensitivity and specificity (93.3% each), and an excellent likelihood ratio (14.0). The proposed physiological mechanism of the significant increase in RRI may be intrarenal vasoconstriction and altered vascular compliance, neurohumoral activation, and venous/back-pressure effects. Similarly, in dehydration-induced hypovolemia, elevated RRI can result from vasoconstriction, decreased vascular compliance, or capillary rarefaction in intrarenal arteries [37,38], However, this reactive mechanism is reversible volume-sensitive [39]. In a study on experimental hypovolemia in dogs, the RRI increased proportionally to the extent of blood loss. In shock and early acute kidney injury, diminished renal perfusion and ischemia lead to higher RRI before Cr levels increase, making it an early hemodynamic marker [40]. In experimental feline hypotension, there was a sharp increase in RRI with strong negative correlation with mean arterial blood pressure. The results confirmed high sensitivity of RRI for recognition of hypovolemia [26]. Similarly, in human medicine, critical care studies have shown that the RRI has high diagnostic and prognostic value in shock [41,42].

CVC diameter was included only in the univariate analysis but not in the final multivariate analysis. The significant reduction in CVC diameter observed in our study is in accordance with several studies on small animals [43,44,45]. Several studies have mentioned the diagnostic significance of CVC diameter in hypovolemia in small animals, as the CVC is highly compliant and decreases in diameter with reduced venous return and blood volume [30,46]. Therefore, point-of-care ultrasound (POCUS) assessment of the caudal vena cava (CVC) and abdominal Ao, alone or as a CVC:Ao ratio, is increasingly used to estimate intravascular volume and fluid responsiveness in small animals [47]. The Ao has received less attention regarding diagnostic significance because it is more muscular and relatively volume-insensitive until more severe hypovolemia [30]. Our findings are also supported by the results of an experiment on hemorrhagic shock in anesthetized dogs, in which there was a significant decrease in both CVC and Ao diameters with marked blood loss (~40 mL/kg) [47].

Although the CVC:Ao ratio showed a non-significant decrease in hypovolemic dogs compared with the controls and was not included in the univariate and multivariate models, previous studies have highlighted its importance in the evaluation of hypovolemia. In the present study, the non-significant change in the CVC:Ao ratio may be attributed to the corresponding decrease in Ao diameter alongside a decrease in the CVC diameter. This explanation was supported by the results of a previous study on experimental hemorrhagic shock in dogs [15]. Conversely, decreased CVC:Ao ratios have been previously associated with volume depletion in dogs with hemorrhagic shock [47] and experimental volume depletion by frequent administration of diuretics [48]. The ratio has also been found to decrease in hypovolemia and increase with blood donation [28]. Moreover, a recent study confirmed that 96% of hypovolemic dogs had a low CVC:Ao ratio [22]. In hospitalized, spontaneously breathing dogs, the CVC:Ao ratio measured at the porta hepatis predicted fluid responsiveness: an area under ROC of 0.88, with a cut-off of 0.83 giving 100% sensitivity and 75% specificity for a ≥15% stroke-volume increase after a mini-bolus [40].

BUN was not included in the final logistic regression model, although it has been reported extensively as a diagnostic indicator of dehydration and hypovolemia [12,49]. Nevertheless, BUN may not be a reliable marker for hypovolemia in dogs because it is influenced by multiple non-volume-related factors, such as protein intake, liver function, and kidney clearance [50]. Consequently, the results of BUN in hypovolemia should be interpreted with caution.

The limitations of the present study should be acknowledged. First, the sample size of the present cases was relatively small, which may have affected the power of the study. However, robust selection criteria for the examined dogs and strong statistical methodology could provide more reliable results. Moreover, previous studies on hypovolemia have been conducted with relatively small sample sizes and obtained outstanding outcomes in dogs [30] and humans [51]. Second, the selected dogs suffered from dehydration, with the exclusion of diseases characterized by organ failure. In our study, we had a single etiology to ensure the homogeneity of the dog group with subsequent reliable results. However, the results cannot be generalized to all cases of hypovolemia. Therefore, further studies are warranted on hypovolemia due to multiple causes to assess ultrasound markers for the early detection of changes. Third, unfortunately, lactate was not measured as a constant marker of hypoperfusion. We relied on the clinical criteria and the echocardiographic findings. Moreover, lactate is affected by several factors, such as hemorrhage. Fourth, we focused mainly on the evaluation of the RRI, not the pulsatility index (PI). However, the RRI is commonly considered more consistent than the PI, as the RRI is more sensitive to changes in renal perfusion and systemic hemodynamics, whereas the PI is more influenced by upstream arterial pulsatility and systemic vascular compliance.

## 5. Conclusions

The results of the present investigation indicate that the noninvasive assessment of RRI via pulsed-wave Doppler ultrasound is a strong diagnostic marker of hypovolemia in emergency and critical care conditions in dogs, especially hypovolemia due to dehydration. Although the diameters of the CVC and Ao, as well as the CVC:Ao ratio, were not included in the final logistic regression model, they may also be reliable candidate markers for early diagnosis. In addition, measurements should be interpreted alongside physical examination and perfusion variables. Further studies are needed to include a large number of dogs with variable clinical conditions associated with hypovolemia to draw stronger conclusions.

## Figures and Tables

**Table 1:** Univariate logistic regression model for ultrasonography and hematobiochemical diagnostic markers in dogs with hypovolemia.

Variables	B	S.E.	Wald	*p*-Value	OR	95% CI	
						Lower	Upper
RRI	5.278	1.464	13.001	0.000	196.000	11.12	34.72
CVC	4.02	1.2	10.8	0.001	51.0	5.2	61.8
Ao	4.1	1.3	10.9	0.001	55.1	5.1	60.1
CVC:Ao ratio	0.539	0.73	.532	0.466	1.714	0.403	7.292
BUN	2.565	0.93	7.502	0.006	13.000	2.074	81.5
Cr	0.055	0.73	0.005	0.941	1.056	0.248	4.499
BUN:Cr ratio	−0.860	0.95	0.806	0.369	0.423	0.065	2.766
PCV%	1.099	0.76	2.089	0.148	3.000	0.676	13.309
TP	0.39	0.725	0.000	0.0999	1.65	0.41	3.2
Platelets	0.085	0.43	0.012	0.821	1.043	0.0251	4.21

RRI, renal artery resistive index; B, regression coefficient; S.E., standard error; OR, odds ratio; CI, confidence interval.

**Table 2:** Final logistic regression model for the diagnostic ultrasonographic diagnostic markers in dogs with hypovolemia.

Variables	B	S.E.	Wald	*p*-Value	OR	95% CI	
						Lower	Upper
RRI	5.278	1.464	13.001	0.000	196.000	11.12	34.72
Constant	−7.917	2.315	11.701	0.001	0.000	-	-

RRI, Renal artery resistive index; B, regression coefficient; S.E., standard error; OR, odds ratio; CI, confidence interval.

## Data Availability

The original contributions presented in this study are included in the article. Further inquiries can be directed to the corresponding authors.
